# Effect of Varying Working Distances between Sandblasting Device and Composite Substrate Surface on the Repair Bond Strength

**DOI:** 10.3390/ma14071621

**Published:** 2021-03-26

**Authors:** Phoebe Burrer, Amanda Costermani, Matej Par, Thomas Attin, Tobias T. Tauböck

**Affiliations:** 1 Clinic of Conservative and Preventive Dentistry, Center for Dental Medicine, University of Zurich, Plattenstrasse 11, 8032 Zurich, Switzerland; amanda.costermani@uzh.ch (A.C.); thomas.attin@zzm.uzh.ch (T.A.); tobias.tauboeck@zzm.uzh.ch (T.T.T.); 2 Department of Endodontics and Restorative Dentistry, School of Dental Medicine, University of Zagreb, Gundulićeva 5, 10000 Zagreb, Croatia; mpar@sfzg.hr

**Keywords:** composite repair, aluminum oxide sandblasting, working distance, microtensile bond strength

## Abstract

This study investigates the effect of defined working distances between the tip of a sandblasting device and a resin composite surface on the composite–composite repair bond strength. Resin composite specimens (Ceram.x Spectra ST (HV); Dentsply Sirona, Konstanz, Germany) were aged by thermal cycling (5000 cycles, 5–55 °C) and one week of water storage. Mechanical surface conditioning of the substrate surfaces was performed by sandblasting with aluminum oxide particles (50 µm, 3 bar, 10 s) from varying working distances of 1, 5, 10, and 15 mm. Specimens were then silanized and restored by application of an adhesive system and repair composite material (Ceram.x Spectra ST (HV)). In the negative control group, no mechanical surface pretreatment or silanization was performed. Directly applied inherent increments served as the positive control group (*n* = 8). After thermal cycling of all groups, microtensile repair bond strength was assessed, and surfaces were additionally characterized using scanning electron microscopy (SEM) and energy dispersive X-ray spectroscopy (EDX). The negative control group reached the significantly lowest microtensile bond strength of all groups. No significant differences in repair bond strength were observed within the groups with varying sandblasting distances. Composite surfaces sandblasted from a distance of 1 mm or 5 mm showed no difference in repair bond strength compared to the positive control group, whereas distances of 10 or 15 mm revealed significantly higher repair bond strengths than the inherent incremental bond strength (positive control group). In conclusion, all sandblasted test groups achieved similar or higher repair bond strength than the inherent incremental bond strength, indicating that irrespective of the employed working distance between the sandblasting device and the composite substrate surface, repair restorations can be successfully performed.

## 1. Introduction

Repair restorations are now widely acknowledged as a valid alternative to total replacement of partially insufficient composite restorations and have gained immense popularity over the last decades [1,2]. Correctly performed, repair restorations may protect healthy tooth structure, reduce the risk of sensitivity and pulpal irritations, and increase the overall longevity of composite restorations [1,2,3,4,5], which is of high benefit for the patient.

A crucial factor for successful repairs is the establishment of sufficient adhesion between the already existing restoration and the repair composite material. The most common causes for failures of composite restorations are fractures, secondary caries, and marginal defects [6]. Therefore, in many cases, repairs need to be performed on aged composite surfaces that often show degradation phenomena [7,8,9,10].

Thus, in order to achieve adequate repair bond strength, mechanical and chemical pretreatment of the existing composite surface is mandatory. Mechanical pretreatments include surface roughening with diamond-coated burs or sandblasting [11,12,13,14,15] in order to increase the surface area and micromechanical retention, allowing for better penetration of the subsequent silane coupling agent and/or adhesive system [4,13]. Therefore, sandblasting of the composite surface is often considered a necessary treatment step of repair procedures [16] and can either be achieved by air abrasion with aluminum oxide particles (Al_2_O_3_) or silica coating [17,18,19]. Although both procedures are accepted, the use of Al_2_O_3_ is more common and has been proven to enhance repair bond strength [13]. Additionally, the use of Al_2_O_3_ over silica-coated particles is preferable, since reduced marginal composite adaptation has been observed for dentin surfaces unintentionally contaminated with silica particles during air abrasion in the course of repair procedures [17].

Correct application of sandblasting devices can be demanding for the practitioner because success depends on the chosen particle size and the applied air pressure required for acceleration and application of particles [20]. In general, both variables are selectable and precisely adjustable in everyday clinical practice. Aluminum oxide powder with a particle size between 25 and 50 µm is ordinarily used, and applied air pressures range from 1.5 bar up to 6 bar depending on the employed sandblasting device [12,13,14,16,17,18,21,22]. However, difficulties during sandblasting procedures may occur when setting the distance between the composite substrate surface and the tip of the sandblasting device, due to a reduced mouth opening or accessibility in posterior regions, which might negatively affect repair bond strength and compromise the long-term result.

Therefore, the aim of the present study was to evaluate this parameter and assess the effect of working distance between the sandblasting device and the composite substrate surface on the composite–composite repair bond strength. In addition, composite surfaces were characterized by scanning electron microscopy (SEM) and energy dispersive X-ray spectroscopy (EDX). The null hypothesis tested was that the applied working distance of the sandblasting device to the composite substrate surface would not affect microtensile repair bond strength.

## 2. Materials and Methods

### 2.1. Specimen Preparation

**Figure 1:**
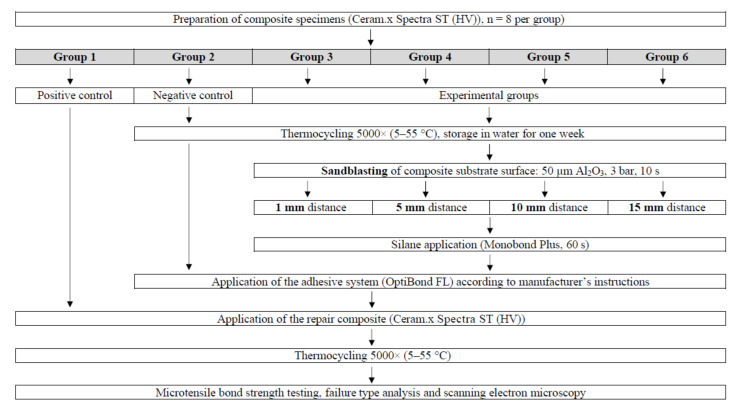
Experimental design.

Figure 1 illustrates the experimental design of the current study. The manufacturer details and the chemical composition of the materials used are provided in Table 1. Forty-eight specimens of a nanohybrid composite with prepolymerized fillers (Ceram.x Spectra ST (HV); Dentsply Sirona, Konstanz, Germany; shade: A4; lot No.: 1907000298) were prepared for this study. This material pertains to contemporary dental resin composites, comprising a modified methacrylate resin matrix filled with ground glass and prepolymerized filler particles. The surfaces of filler particles are typically coated with a silane coupling agent that enables their bonding to the methacrylate resin. The composite material is rendered photosensitive by camphorquinone and tertiary amine, enabling it to set on-demand in clinical work by illumination using blue light [23].

To build the substrate composite complex, first, a 2 mm thick resin increment was adhered to a carrier usually used for scanning electron microscopy (Wenka, Karl Wenger SA, Courgenay, Switzerland) with the help of a custom-made cylindrical silicone mold of 16 mm diameter. This increment served as a base for the three following resin composite increments of 1.5 mm thickness, which were applied by using smaller cylindrical silicone molds with a diameter of 10 mm. Each increment was levelled with a PTFE-roller (CompoRoller 5300; KerrHawe, Bioggio, Switzerland) to produce a flat surface. Photopolymerization was performed for 20 s with a blue LED light-curing unit (SmartLite Pro; Dentsply Sirona, Konstanz, Germany). A continuous output irradiance of approximately 1033 mW/cm^2^ was ensured throughout the whole study by using a calibrated FieldMax II-TO power meter (Coherent, Santa Clara, CA, USA) [10].

In the positive control group, composite repair was immediately performed by placing another three 1.5 mm thick repair composite increments onto the composite substrate surface, representing the inherent incremental bond strength [14]. Groups 2–6, however, were first polished with 4000-grit silicon carbide grinding paper (Buehler-Met II; Buehler, Esslingen, Germany) in a polishing machine (Planopol-2; Struers, Ballerup, Denmark) while being constantly water-cooled. Afterwards, the specimens were submitted to artificial aging by thermal cycling (5000 cycles, 5–55 °C, dwell time: 20 s, transfer time: 10 s) and storage in tap water for one week. Composite specimens representing the negative control group (group 2) were then repaired without further mechanical or silane pretreatment. In this group, the adhesive system (OptiBond FL; Kerr, Orange, CA, USA) was directly applied on the composite substrate surface, strictly following the manufacturer’s instructions for use, and light cured for 20 s.

### 2.2. Aluminum Oxide Sandblasting

In contrast to the positive and negative control groups, specimens of experimental groups 3–6 received additional mechanical pretreatment by sandblasting after the artificial aging process. For this purpose, a custom-made sandblasting tower was manufactured. Composite specimens were positioned on a height-adjustable screw device that enabled the exact adjustment of defined distances between the composite surface to the tip of the sandblasting device (MicroEtcher II; Danville Materials/Zest Dental Solutions, Carlsbad, CA, USA). The nozzle tip in turn was guided through a broadened support surface to ensure homogenous and perpendicular sandblasting of the composite surface. Air pressure of 3 bar was controlled during the sandblasting procedure by means of a manual pressure gauge. Sandblasting was then performed for 10 s using aluminum oxide powder with a particle size of 50 µm (RondoFlex Preparation Powder; KaVo, Biberach/Riss, Germany; lot. No.: 2018091002) from defined working distances of 1 mm (group 3), 5 mm (group 4), 10 mm (group 5), and 15 mm (group 6). Remnants of Al_2_O_3_-particles were air blown away. Afterwards, composite specimens of groups 3–6 were treated with a silane coupling agent (Monobond Plus; Ivoclar Vivadent, Schaan, Liechtenstein; lot No.: Y24458) that was applied for 60 s with a microbrush in a thin layer, followed by application of the adhesive system (OptiBond FL; Kerr, Orange, CA, USA) prior to repair.

### 2.3. Application of Repair Composite

All groups received a composite repair restoration and were built up with another three resin composite increments of 1.5 mm thickness using the same nanohybrid composite as for the first composite buildup, but of a different shade (Ceram.x Spectra ST (HV); Dentsply Sirona, Konstanz, Germany; shade: A1; lot No.: 1906000385). The positive control group (group 1), representing the inherent incremental bond strength, received immediate repair, whereas the negative control group (group 2) was repaired without additional pretreatment by air abrasion and silane application. Analogous to the first composite buildup, composite increments for the repair buildup were applied by using further silicone molds of 10 mm diameter. Again, each increment was photoactivated for 20 s.

Specimens of all groups were then exposed to an artificial aging procedure by thermal cycling (5000 cycles, 5–55 °C, dwell time: 20 s, transfer time: 10 s) prior to microtensile bond strength testing.

### 2.4. Microtensile Bond Strength Test

To determine repair bond strength, specimens of all groups were subjected to a standard bond strength testing procedure previously described in detail [10,24]. Briefly, specimens were cut lengthways and crossways in a precision cutting machine (Struers Accutom-50; Struers GmbH, Ballerup, Denmark) with a diamond cut-off wheel (M1D10; Struers GmbH, Ballerup, Denmark) under constant water cooling. Nine rectangular sticks of approximately 0.9 × 0.9 × 9 mm^3^ from the center of each specimen were obtained, and the exact dimensions of the resulting sticks were measured with a digital metric measuring gauge (Coolant Proof Micrometer 293-230-30; Mitutoyo AG, Urdorf, Switzerland) to calculate the cross-sectional bonding area. The obtained sticks were then adhered to a sandblasted (50 µm Al_2_O_3_) microtensile bond strength jig with cyanoacrylate glue (Superglue No. 1733-2000; Renfert, Hilzingen, Germany). For microtensile bond strength testing, a universal testing machine (ZwickRoell Z010; ZwickRoell GmbH, Ulm, Germany) was used to load the sticks under tension until failure. The crosshead speed was set to 1 mm/min, and the load (N) recorded when sticks failed was divided by the cross-sectional bonding area (mm^2^) to obtain microtensile bond strength in MPa.

### 2.5. Assessment of Failure Mode

After microtensile bond strength testing, failure modes of all tested sticks were determined using an optical microscope (Stemi 1000; Fisher Scientific, Reinach, Switzerland) at 25× magnification. Depending on the type of fracture, failures were classified as adhesive when failure occurred between substrate and repair composite, as cohesive when failure occurred within the substrate or within the repair composite, or judged as mixed failure when a combined adhesive and cohesive failure occurred.

### 2.6. SEM and EDX Analysis of the Substrate Surfaces

In order to characterize the surface morphology of the composite substrate surfaces after aluminum oxide sandblasting, additional specimens were prepared. Composite specimens were dried and sputter-coated with gold to achieve a layer of 10 nm (Sputter coater Safematic CCU-010; Safematic GmbH, Zizers, Switzerland), and scanning electron microscope (SEM) images (GeminiSEM 450; Zeiss, Oberkochen, Germany) were taken at a working distance of 8–9 mm at 5000× magnification (15 kV).

In addition, energy dispersive X-ray spectroscopy (EDX-MaxN; Oxford instruments, High Wycombe, UK) was performed to examine the chemical composition of the substrate surfaces. Five scan plots were taken at regular distances of 15 µm along a scanning line in the upper and lower area of the SEM image (10 kV) at 5000× magnification, and element analysis by the Point-and-ID-method was assessed.

### 2.7. Statistical Analysis

Failure of specimens occurring prior to microtensile bond strength testing was classified as pretest failure and its bond strength was set to 0 MPa [25]. The Shapiro–Wilk test and Levene’s test were performed to test the data for normality of distribution and homogeneity of variances. Due to the fact that the results were not normally distributed, nonparametric tests were employed in the statistical analysis. Data were analyzed using Kruskal-Wallis tests and resulting p-values were corrected for multiple testing according to Bonferroni. All statistical analyses were performed using SPSS version 26 (IBM Corp., Armonk, NY, USA) with an overall level of significance of α = 0.05.

## 3. Results

### 3.1. Microtensile Repair Bond Strength

**Figure 2:**
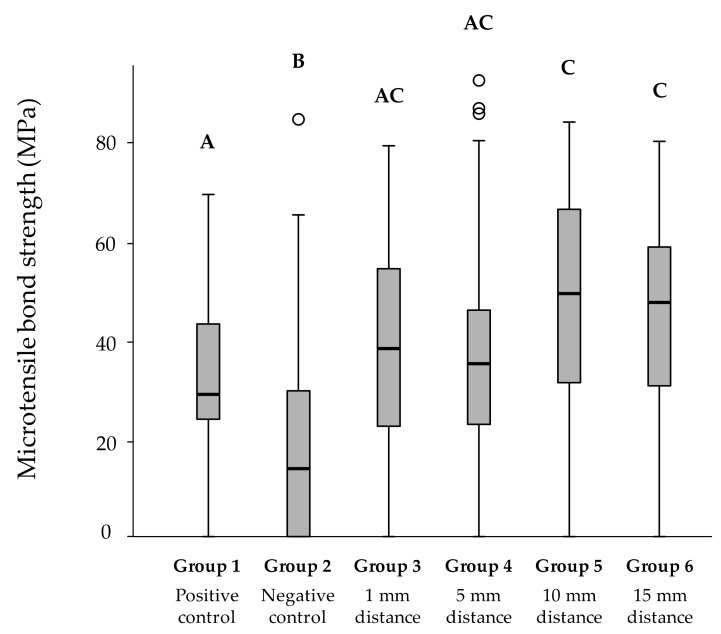
Microtensile repair bond strengths (MPa) of composite surfaces after sandblasting from varying working distances. Significant differences between groups are indicated by different letters (*p* < 0.05). Within each boxplot, the median is represented by a horizontal bold black line. The 25% and 75% data quartiles are shown as boxes, and the whiskers mark the 1.5 × interquartile range (IQR) at the 25th and 75th percentile of each group. The outliers of groups 2 and 4 are shown as circles.

Figure 2 illustrates the microtensile repair bond strengths of the composite substrate surfaces pretreated with aluminum oxide sandblasting from the different working distances. The negative control group (group 2: 19.1 MPa ± 13.0) reached the significantly lowest microtensile bond strength of all groups (*p* < 0.05). Repair bond strength of the positive control group (group 1: 32.2 MPa ± 4.8) did not differ significantly from composite surfaces pretreated with aluminum oxide particles at a distance of 1 mm (group 3: 39.1 MPa ± 8.5) or 5 mm (group 4: 37.8 MPa ± 9.5). However, significantly higher bond strengths were found for group 5 with a sandblasting distance of 10 mm (group 5: 47.5 MPa ± 12.1) and group 6 with a distance of 15 mm (group 6: 43.9 MPa ± 10.3), compared to the positive control group (group 1). No significant differences in repair bond strengths could be observed within the experimental groups (groups 3–6) with working distances from 1 mm to 15 mm between composite substrate surface and tip of the sandblasting device.

### 3.2. Failure Mode Distribution

**Figure 3:**
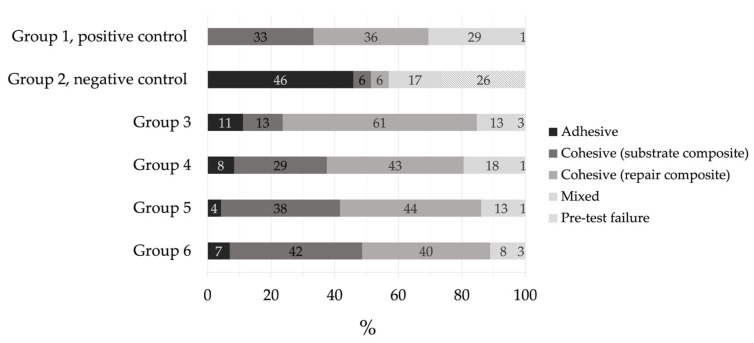
Percentage (%) of failure mode distribution per group.

Figure 3 depicts the failure mode distribution of all groups of the current study assessed at 25× magnification with an optical light microscope. In the majority of groups (groups 1, 3–5), the most frequent failure mode was cohesive within the repair composite. In group 6, however, cohesive failures in the substrate composite were most frequently observed. The highest percentage of pretest failures was found in the negative control group (group 2).

### 3.3. SEM Analysis of the Substrate Surfaces

**Figure 4:**
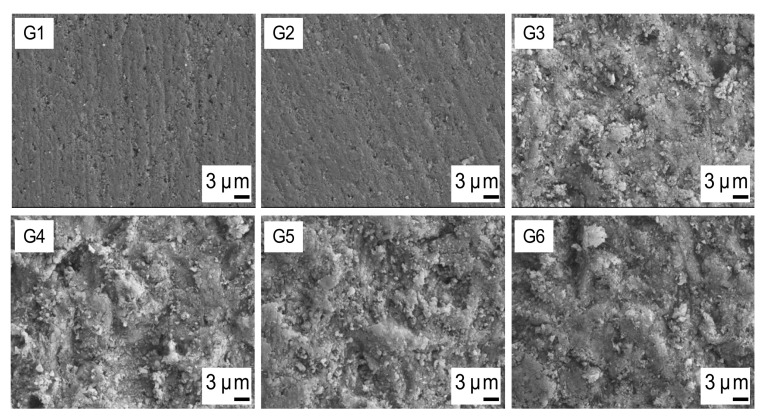
Scanning electron microscopy (SEM) images (5000× magnification) of the composite surfaces of all groups. G1: positive control group; G2: negative control group; G3–G6: composite substrate surfaces sandblasted from working distances of 1 mm (G3), 5 mm (G4), 10 mm (G5), and 15 mm (G6).

Micromorphological SEM images of the composite substrate surfaces are shown in Figure 4. In G1, the untreated composite surface is visible, whereas in G2 the aged composite surface without mechanical pretreatment is presented, both showing similar surface topography. G3–G6 of Figure 4 display the aged composite surfaces sandblasted from the varying working distances of 1 mm (G3), 5 mm (G4), 10 mm (G5), and 15 mm (G6). They exhibit similar surface structures among themselves, but they display a more irregular topography than the untreated positive control group (G1) and the solely aged composite surface of the negative control (G2). In addition, analysis by energy dispersive X-ray spectroscopy revealed a similar chemical composition of groups 3–6 with no prominently high signals for aluminum. Table 2 presents the percentages of aluminum detected on the substrate surfaces based on the EDX element analysis by the Point-and-ID-method.

## 4. Discussion

Even though repair of composite restorations has become increasingly popular and can be regarded as an established treatment procedure, there are still uncertainties left regarding the setting parameters when it comes to air abrasion by sandblasting. It is of particular clinical importance for the practitioner to know details about the application when performing repairs using sandblasting techniques. The present study is, to the best of the authors’ knowledge, the first to examine the influence of the parameter of working distance between the composite surface and the tip of the sandblasting device on the repair bond strength. Our results revealed similar or higher repair bond strength after sandblasting compared to the inherent incremental bond strength (positive control, group 1), but no significant differences in repair bond strength could be observed within groups 3–6 with varying working distances of 1–15 mm between the composite surface and the tip of the sandblasting device. The SEM examination and EDX analysis confirmed these findings, revealing similar irregularly roughened substrate surfaces of similar element composition after air abrasion from different working distances (groups 3–6). Thus, the tested null hypothesis could not be rejected.

In the present study, cohesive failures in the repair composite were the predominant failure mode in most of the experimental groups, followed by cohesive failures in the substrate composite. The small number of adhesive failures in all groups except the negative control group (group 2) is noticeable and might be explained by degradation processes in the composite material due to aging by thermocycling, resulting in compromised mechanical properties [8,26]. In agreement with the findings of the present investigation, a study of Valente et al. [15] also revealed more cohesive failures for aged composite compared to fresh composite.

Previous research has mainly focused on the type of pretreatment and many attempts have been made to establish a standard repair protocol, which is challenging due to the many different parameters and application steps that have to be performed. Uncertainties remain regarding the details about the air abrasion procedure by sandblasting, such as applied air pressure, particle size, or sandblasting distance. However, there exists a consent in literature that the fundamental basis of repair restorations is the process of increasing the surface roughness of the composite substrate. It has been proven by numerous studies that the rougher the composite surface, the higher the repair bond strengths that can be achieved [10,12,13,15,18,19,27,28], with air abrasion being more effective than roughening by diamond burs [11,12,13,18]. Indeed, the present results emphasize the necessity of surface roughening for mechanical interlocking, which results in significantly higher repair bond strengths compared to the negative control group without mechanical pretreatment (group 2).

Although the results of the present study showed that the distance between the composite surface and the tip of the sandblasting device had no significant influence on the composite–composite bond strength, larger working distances of 10 mm or 15 mm yielded numerically higher repair bond strength than distances of 1 mm or 5 mm. The optimal interlocking between substrate and repair composite material at larger distances might be attributed to a greater scattering at larger sandblasting distances, enabling a more homogenous roughening of the composite surface. However, the patient’s mouth opening, or limited accessibility might hamper the adjustment of these larger distances. Furthermore, with increasing distances, preciseness becomes more demanding. On the other hand, with regard to aluminum oxide particles applied from smaller working distances of 1–5 mm to the substrate composite material, creation of focal points could occur and negatively affect repair bond strengths.

In certain cases, it might also seem difficult to get as close as 1 mm or 5 mm to the substrate composite. However, the results of the present study showed no significant difference when sandblasting was performed from these working distances compared to the inherent incremental bond strength. Indeed, many studies on composite repair performed sandblasting with air abrasion particles at a working distance of 5 mm [12,13,27,29], while others operated at a distance of 10 mm [14,18], 20 mm [21] or even 30 mm [22]. In this context, the factors of size, speed, and type of the applied abrasive particles also correlate with material removal of the substrate composite and affect the surface morphology [11,20]. The chosen particle size of 50 µm [11,12,13,14,17] and application time of 10 s [13,29] are thus in accordance with other studies examining repair procedures.

Additionally, the angulation of the tip of the sandblasting device could potentially influence repair bond strength. It seems advisable to position the nozzle perpendicular to the substrate surface to achieve the best results [14]. Therefore, in the present study, a custom-made distance holder was used to maintain both the chosen working distance and angulation during sandblasting. However, implementation in everyday clinical practice can be difficult and should also be considered when adjusting the distance for sandblasting.

Similar to other studies on repair bond strengths [13,29], in the current investigation, bond strength values between 38 MPa (group 4: 5 mm working distance) and 48 MPa (group 5: 10 mm working distance) were achieved. As these values can be considered adequate for sufficient bonding [20], our results indicate that repair procedures can be recommended regardless of the employed working distance. Additionally, silane application and application of an adhesive system may have further contributed to adhesion between the substrate and repair composite [7,11,22,27,30,31,32].

Correlating with the achieved repair bond strengths, SEM images showed comparable irregular surfaces after sandblasting, exhibiting similar micro-retentive patterns [21]. These structural similarities may confirm the assumption that an increase of repair bond strengths may be due to a better micromechanical interlocking between substrate and repair composite after sandblasting with aluminum oxide particles. Furthermore, results of the energy dispersive X-ray spectroscopy showed a similar chemical composition within experimental groups 3–6. Although slightly more aluminum components were detected for groups 3–6, compared to the positive control group, results were negligible as particles were homogenously distributed on the composite surfaces, indicating that no significant incorporation or retention of aluminum particles occurred, which is in line with the findings of Rathke et al. [11]. Thus, it seems likely that the higher surface roughness of groups 3–6 in comparison with the positive control group can be attributed to the effect of sandblasting from various working distances, resulting in similar or higher bond strength values compared to the inherent incremental bond strength.

The investigation of only one resin composite material and one type of sandblasting powder can be regarded as potential limitations of the present study. Therefore, comparisons with other materials should be drawn with caution. However, aluminum oxide sandblasting for microroughening of composite surfaces to improve adhesion is largely accepted and represents a basic tool of resin composite pretreatment, which is corroborated by the results of the present study. Further studies should investigate the interaction of different materials and sandblasting powders to gain more consensus about the setting parameters of repair protocols.

Therefore, within the limitations of the present study, it can be concluded that the working distance between the sandblasting device and the substrate surface had no influence on the composite–composite repair bond strength, and inherent incremental bond strength was achieved or exceeded by all groups pretreated by air abrasion. The results thus indicate that repair restorations can be successfully performed irrespective of the applied distance between the substrate surface and the sandblasting device.

## Figures and Tables

**Table 1:** Manufacturers’ information about the main materials used in the present study.

Product	Composition	Lot no.	Manufacturer
Ceram.x Spectra ST (HV)	Matrix: methacrylic modified polysiloxane nanoparticles, dimethacrylate resin, ethyl-4-(dimethylamino)benzoate Filler: spherical, pre-polymerized SphereTEC fillers (particle size ≈ 15 μm), non-agglomerated barium glass, CQ ^1^, ytterbium fluoride Filler content: 78–80 wt%, 60–62 vol%	A4: 1907000298 A1: 1906000385	Dentsply Sirona, Konstanz, Germany
Monobond Plus	Alcohol, silane methacrylate, 10-MDP ^2^, phosphoric acid methacrylate, sulphide methacrylate	Y24458	Ivoclar Vivadent, Schaan, Liechtenstein
OptiBond FL	Primer: BHT ^3^, CQ, ethanol, GPDM ^4^, HEMA ^5^, PAMM ^6^, water Adhesive: Bis-GMA ^7^, CQ, GDM ^8^, HEMA, ODMAB ^9^, barium aluminoborosilicate, Na_2_SiF_6_, fumed silicon, dioxide, gamma-methacryloxypropyltrimethoxysilane	Primer: 6284132 Adhesive: 6496643	Kerr, Orange, CA, USA

^1^ CQ: camphorquinone; ^2^ 10-MDP: 10-methacryloyloxydecyl dihydrogen phosphate; ^3^ BHT: butylhydroxytoluen; ^4^ GPDM: glycerol phosphate dimethacrylate; ^5^ HEMA: 2-hydroxylethyl methacrylate; ^6^ PAMM: phthalic acid monomethacrylate; ^7^ Bis-GMA: bisphenol-A-glycidyl-dimethacrylate; ^8^ GDM: glycerol dimethacrylate; ^9^ ODMAB: 2-(Ethylhexyl)-4-(dimethylamino)benzoate.

**Table 2:** Energy dispersive X-ray spectroscopy (EDX) weight percentages (wt%) of aluminum components on the composite substrate surfaces per group.

Group	Percentage of Aluminum
Group 1, positive control	2.5
Group 2, negative control	2.7
Group 3	4.1
Group 4	3.7
Group 5	4.0
Group 6	3.9

## Data Availability

The datasets generated and analyzed during the current study are available from the corresponding author on reasonable request.
